# Climate change: A threat towards achieving ‘Sustainable Development Goal number two’ (end hunger, achieve food security and improved nutrition and promote sustainable agriculture) in South Africa

**DOI:** 10.4102/jamba.v9i1.350

**Published:** 2017-02-27

**Authors:** Shingirai S. Mugambiwa, Happy M. Tirivangasi

**Affiliations:** 1 Department of Sociology and Anthropology, University of Limpopo, South Africa

## Abstract

This article aims to assess the impacts of climate change towards the achievement of Sustainable Development Goal number two (SDG 2) as well as examining the poverty alleviation strategies by subsistence farmers in South Africa. Widespread hunger and poverty continue to be among the most life-threatening problems confronting mankind. Available statistics show that global poverty remains a serious challenge around the world. Across the globe, one in five people lives on less than $1 a day and one in seven suffers from chronic hunger. Similarly, the developing world is adversely affected by poverty and hunger. In the sub-Saharan Africa, research has revealed a higher prevalence of hunger, malnutrition, poverty and food insecurity. SDG 2 focuses more on eliminating hunger and promoting sustainable agriculture. The study employed an exploratory design and a qualitative method. Snowball sampling was used in selecting relevant sources which led the researchers to other research work on the same field through keywords and reference lists. The researchers employed discourse analysis to analyse data. The study discovered that there are numerous potential effects climate change could have on agriculture. It affects crop growth and quality and livestock health. Farming practices could also be affected as well as animals that could be raised in particular climatic areas. The impact of climate change as well as the susceptibility of poor communities is very immense. The article concludes that climate change reduces access to drinking water, negatively affects the health of people and poses a serious threat to food security.

## Introduction and background

Poverty and hunger are a common phenomenon to mankind, especially in the developing world (Rasul 2016). Consistently, widespread hunger and poverty continue to be among the most life-threatening problems confronting mankind (Cobbinah, Erdiaw-Kwasie & Amoateng 2015). Available statistical data show that Western countries continue to develop in all spheres of life, while the poor south becomes stuck in the swamps of hunger and malnourishment. In spite of the economic boom in the Western world, many countries around the globe still face the problem of poverty. Various studies reveal that one in five people live on less than $1 a day, whereas one in seven are suffering from hunger (World Bank 2011). Worldwide, the number of people living on less than $370 annual income stands at about 1.3 billion (World Hunger Education Service 2011; Van der et al. 2012). Consequently, the reason for the poor south to remain stuck in the chains of poverty and hunger is partly because of climate change as they largely depend on subsistence economy and natural resources for their survival. It is argued that climate change results in depleted water reservoirs and drinking water, which will consequently affect the health and food security of poor folks in underdeveloped communities in many countries in Africa, Latin America and Asia (IPCC 2014; OECD 2014).

Aljareo (2014) postulated that countries all over the world are subject to climate change and South Africa is not an exception in that regard. Calzadilla et al. (2014) were of the opinion that poverty alleviation strategies and sustainable agriculture are seriously hampered by climate change in South Africa. Calzadilla et al. (2014) and Maponya and Mpandeli (2012) noted that climate change will result in high temperature and less rainfall in South Africa. This will have a negative impact on food production and the productivity of cropland (Calzadilla et al. 2014). Further, this will affect the food prices on local markets and international trade patterns. As such South Africa needs to craft climate change policy that will help in dealing with environmental and socio-economic effects of climate change. This policy should aim to support sustainable development achievements, which South Africa has considered as priority in decision-making processes.

This is important because hunger, poor nutrition and food insecurity are serious challenges in South Africa. Though South Africa has adequate food for its population, it can be noted that most poor households remain vulnerable to increasing food prices which prevent many people from accessing safe and healthy food (Hosken 2013). Furthermore, majority of people in South Africa as in other developing countries rely heavily on agriculture for survival and will, therefore, be vulnerable to climate change. In this regard, one can note that climate change has significant effects on food security and malnutrition. In turn, achieving Sustainable Development Goal number two (SDG 2) will be a serious challenge owing to the threats posed by climate change.

This article focuses on the impacts of climate change towards the achievement of SDG 2 as well as examining the poverty alleviation strategies by subsistence farmers in South Africa. The study provides knowledge on the impacts of climate change as well as helping to inform the various stakeholders in the government and humanitarian groups on the ways of enhancing the farmer’s adaptation strategies to climate change. This article will discuss the SDGs, modernisation theory, methodology used, the results and discussion. Subsequently, the conclusion and recommendations are also given.

## Sustainable development goals

There are 17 SDGs and 169 targets. The SDGs have been intended to go beyond the millennium development goals (MDGs) and to provide a comprehensive vision and framework for the evolution of all countries in future (Joshi, Hughes & Sisk 2015). The adoption of SDGs was a result of the failure of countries to achieve the MDGs. The aim of the MDGs was to end various ills in developed and developing countries in order to help people achieve their full potential. However, by the year 2015 which was the deadline for the MDGs, many countries had not achieved the agreed upon goals (Millennium Development Goals Report 2015). Even though some countries could not achieve the MDGs by the deadline, that is, 2015, there is a variety of success stories that have been recorded in various countries. Millennium Development Goals Report (2015) showed that globally, poverty levels decreased over the past two decades, with many countries achieving MDG number one 5 years prior to the deadline. The number of people living on less than a dollar a day globally fell from 36% in 1990 to about 15% in 2011. United Nations estimates reveal that the global poverty rate fell further to 12% in 2015 (Millennium Development Goals Report 2015).

On the contrary, there was no change on sub-Saharan Africa’s 1990 poverty rate until after 2002 (The Millennium Development Goals Report 2014). Despite some noticeable change over the past decade, the region continues to lag behind the rest of the world. Approximately 40% of sub-Saharan Africa still lived in dire poverty in 2015 (Millennium Development Goals Report 2015). The world’s extremely poor people are distributed unevenly all over the world. Approximately 80% of the global total of extremely poor people were found in Southern Asia and sub-Saharan Africa in 2014 (The Millennium Development Goals Report 2014).

The United Nations General Assembly agreed to adopt the report of the Open Working Group on Sustainable Development Goals in 2014 as the basis for integrating the SDGs into the post 2015 development agenda. SDG 2: ‘End hunger, achieve food security and improved nutrition, and promote sustainable agriculture’ like other SDGs has a target that is aimed at the year 2020 and 2030. Rasul (2016) noted that sustainable agricultural development is important in order to improve food security and environmental issues. However, achieving SDG 2 requires the policy-makers to be cognisant of natural biophysical processes and economic and social processes because there is no sufficient evidence on how to improve food security and nutrition strategies (Joshi et al. 2015) in the midst of climate change. Climate change has various effects on agriculture, for instance, it has impact on crop growth, quality and livestock health (Bryan et al. 2013). In turn, these could affect the supply and demand of agricultural products on the market resulting in increased cost of living. Furthermore, change in climate may also result in depleted water sources and unmet water needs for agriculture. Moreover, the need for irrigation increases because of temperature increases and unreliable rainfall events resulting from global warming (Sapkota et al. 2015).

## Sustainable Development Goal number two

SDG 2 stipulates the following:

2.1 By 2030, end hunger and ensure access by all people, in particular the poor and people in vulnerable situations, including infants, to safe, nutritious and sufficient food all year round

2.2 By 2030, end all forms of malnutrition, including achieving, by 2025, the internationally agreed targets on stunting and wasting in children under 5 years of age, and address the nutritional needs of adolescent girls, pregnant and lactating women and older persons

2.3 By 2030, double the agricultural productivity and incomes of small-scale food producers, in particular women, indigenous peoples, family farmers, pastoralists and fishers, including through secure and equal access to land, other productive resources and inputs, knowledge, financial services, markets and opportunities for value addition and non-farm employment

2.4 By 2030, ensure sustainable food production systems and implement resilient agricultural practices that increase productivity and production, that help maintain ecosystems that strengthen capacity for adaptation to climate change, extreme weather, drought, flooding and other disasters and that progressively improve land and soil quality

2.5 By 2020, maintain the genetic diversity of seeds, cultivated plants and farmed and domesticated animals and their related wild species, including through soundly managed and diversified seed and plant banks at the national, regional and international levels, and ensure access to and fair and equitable sharing of benefits arising from the utilization of genetic resources and associated traditional knowledge, as internationally agreed

2.a Increase investment, including through enhanced international cooperation, in rural infrastructure, agricultural research and extension services, technology development and plant and livestock gene banks in order to enhance agricultural productive capacity in developing countries, in particular least developed countries

2.b Correct and prevent trade restrictions and distortions in world agricultural markets, including through the parallel elimination of all forms of agricultural export subsidies and all export measures with equivalent effect, in accordance with the mandate of the Doha Development Round

2.c Adopt measures to ensure the proper functioning of food commodity markets and their derivatives and facilitate timely access to market information, including on food reserves, in order to help limit extreme food price volatility (ICSU & ISSC 2015: p. 6)

## Theoretical framework

### Modernisation theory

Yeh (1989) noted that modernisation theory emerged in the late 1950s and early 1960s from America’s new position of international domination and the need to solve the problems of underdeveloped countries. According to Joshi (2005), modernisation theory posits that Third World countries are underdeveloped because of their historical failure to change and adopt new technology. The claim attributes that the Third World was left behind by the First World and that it must catch up through adopting the same measures undertaken by the First World countries. The modernisation theory reveals that primitive, anti-productive methods and social practices must be eliminated in order for the developing countries to change and develop. In addition, problems such as poverty, war, famine, economic stagnation and the failure to catch up with the First World technologically impede development. Huntington (1968a:52; in Tipps 1973) proclaimed that ‘Modernization is a complex process involving changes in all areas of human mind and movement’. Furthermore, Eisenstadt (1966:43; in Tipps 1973) argued that:

modernization is characterized by two features, which are one, a type of change (structural differentiation) and the other a type of response to change (the capacity of institutions to absorb ‘continually changing problems and demands’). (p. 23)

Hence, climate change can also be considered as a barrier to ‘catching up’ with the First World in agricultural technology and eradication of hunger and malnutrition as well as achieving SDG 2. Climate change is a worldwide concern but the effect is more intense in the Third World because of the state of development and technological advancement. Hence, catching up with the First World will always be a serious challenge in the midst of climate change.

## Methodology

### Design

This study employed an exploratory design and a qualitative method. Exploratory design is one of the most useful (and appropriate) research designs for projects that address a subject about which there are high levels of uncertainty and ignorance as well as when the problem is not very well understood (Bryman 2012).

### Sampling

#### Snowball sampling

Snowball sampling is a non-probability sampling method that involves data sources nominating other potential data sources to be used in the research. Hence, it is a method based on referrals from initial subjects to generate additional subjects (Bryman 2012). For the current study, the researchers employed snowball sampling in selecting relevant sources which would lead them to other research work on the same field through keywords and reference lists.

### Data analysis

The researcher employed discourse analysis. It refers to the practises of talking and writing (Woodilla 1998), which brings text into being through the production, dissemination and consumption of text. Therefore, the goal of discourse analysis is to explore the relationship between discourse and reality as well as interpretation of hidden meaning (Bondarouk & Ruel 2004). The researcher analysed the data collected from the Internet, databases, newspapers, journal articles and books. In so doing, the relationship between discourses, that is, other authors’ findings on climate change in South Africa and reality pertaining to climate change as a threat to achieving SDGs was established.

## Results and discussion

The researcher utilised articles from various databases and hand searches over a period of 3 weeks. However, most of these articles were not deemed necessary. Ziervogel et al. (2014) found out that in South Africa, the issue of climate change is becoming a serious concern and that urgent socio-economic developmental needs and identifying ways to adapt to current as well as future climate variability and change are critical to South Africa.

Financial and Fiscal Commission report (2015) noted that climate change has serious impacts on South African poor households which render them more vulnerable. The commission’s report that was carried out in collaboration with FANRPAN looks at the micro level impacts of climate change at household levels. It uses the household vulnerability index which is a statistical tool used to quantify household vulnerability that results from shocks such as climate change to identify vulnerable households. The study sites were Nkonkobe Municipality in the Eastern Cape and Thulamela Municipality in the Limpopo Province. The climate change impact model results reveal that crop yields will be heavily affected by climate change. Moreover, the study reveals the most vulnerable provinces in South Africa; the report discovered that both Limpopo and Eastern Cape were vulnerable, though the vulnerability of the Eastern Cape Province was more intense than that of the Limpopo Province. Also, it was discovered that the incidence of vulnerability is higher among households with less livelihood capital assets, for instance human, physical, financial, social and natural.

### Climate change and ending hunger as well as achieving food security and improved nutrition

Sanbi (2013) noted that, in South Africa, climate change is predicted to escalate the extent and prevalence of droughts, high temperatures and rainfall variability. This is likely to affect food systems which will in turn compromise food availability and utilisation resulting in food insecurity. Climate change associated rainfall forecasts remain uncertain; for example, Western and Northern Cape provinces could become drier, whereas Central and Eastern plateau could experience increased rainfall. Without effective adaptation, such change in rainfall distribution possibly reduces crop yields, especially if temperatures are higher. As a result, there would be intense food shortages and in turn food prices would increase and vulnerable households will suffer the most.

One of the main objectives of SDG 2 is to end hunger and malnutrition. Consequently, if the country experiences food shortages as a result of climate change, achieving the SDG target will be difficult. Few people will afford basic foodstuffs considering the socio-economic situation in South Africa as a developing country. SDG 2 target is that by 2030, hunger should have been defeated and malnutrition should have been conquered. SDG 2 target ‘one’ says, by 2030 globally, nations should be able to end hunger and ensure access to safe, nutritious and sufficient food by all people in vulnerable situations, including infants, throughout the year. Nevertheless, in the midst of climate change, ensuring access to safe, nutritious and sufficient food to all people will indeed be a pipe dream.

Consistently, Edame et al. (2011) discovered that climate change will deteriorate the living conditions of farmers, fishers and forest-dependent people who were already in need of food. Rural communities reliant on agriculture will face an instantaneous risk of crop failure and loss of livestock. Consequently, hunger and malnutrition will increase because most of these people earned their income from agricultural products. Loosing livestock and crop failure will reduce their source of food, and malnutrition will increase remarkably such that as a result of climate change, achieving SDG in South Africa will be a fantasy. Lisa Dreier, who is the head of Agriculture and Food Security Initiatives, argued that:

hunger can be eliminated within this lifetime, if we create better opportunities for farmers and focus on the needs of undernourished groups. Sustainability means using fewer natural resources to produce food and reducing food waste and loss. Improved nutrition means reducing both hunger and obesity through improved education, and access and availability of quality foods. (World Economic Forum 2015:1)

In this regard, South Africa cannot create better opportunities if farming activities are disrupted by climate change. Also, South Africa is a developing country with majority of subsistence farmers in the rural areas still utilising natural resources to produce food, which Lisa Dreiser condemned. Hence, ending hunger to meet the objectives of SDG 2 will be quite challenging for South Africa.

### The impact of climate change on achieving sustainable agriculture

Achieving sustainable agriculture as one of the targets of SDG 2 is also seriously threatened by climate change. This is because in the presence of climate change, agricultural production will be seriously threatened. As a result, achieving SDGs by the deadline in South Africa will be a challenge. UCT (2008) proclaimed that South Africa has a twofold agricultural economy that includes well-developed commercial farming and small-scale farming. There are seven climatic regions that range from semi-desert to Mediterranean conditions. The distribution of rainfall is largely uneven. The average annual rainfall is 450 mm per year and it is well below the world’s average of 860 mm. Evapo-transpiration is moderately high, and possible evaporation estimated at 1500 mm per year, resulting in only 8.5% run-off and a joint run-off of 42 mm per year equated with the average for the entire continent which is 139 mm per year. Nonetheless, the run-off in the country is not only exceedingly low, but also varies from year to year as well as region to region.

These statistics seriously disrupt agricultural production. The country’s annual rainfall among other mentioned natural aspects is half the world average which is a clear indication that the country receives little rainfall than expected. The little rainfall that the country receives affects the intention of the country to expand productivity in order to meet the SDG deadline. SDG number 2.3 states that:

by 2030, double the agricultural productivity and incomes of small-scale food producers, in particular women, indigenous peoples, family farmers, pastoralists and fishers, including through secure and equal access to land, other productive resources and inputs, knowledge, financial services, markets and opportunities for value addition and non-farm employment. (ICSU & ISSC 2015:6)

Remarkably, Gbetibouo and Hassan (2005) studied seven field crops, namely maize, wheat, sorghum, sugarcane, groundnut, sunflower and soybean. They discovered that both season and location were vital in establishing the probable effects of climate change on crop production. Moreover, they found that temperature increases had a negative effect and precipitation increases had a positive effect on production and output. Therefore, if the climatic conditions continue in the same manner, it will be a serious challenge for South Africa to achieve the targets of SDG 2.

UCT (2008) asserted that in South Africa, the agricultural sector is the spine of the country’s economy. Existing evidence suggests that climate change could likely lead to a fall of about 1.5% in the country’s gross domestic product (GDP) by 2050. The fall is unevenly equal to the aggregate annual foreign direct investment in South Africa presently. Furthermore, climate change has serious penalties on other economic sectors that are directly or indirectly associated with the agricultural sector. Thus, instability may likely cause other sectors to be extremely unstable through backward and forward links, more especially for economies that are directly reliant on exports of primary commodities.

If the economic sectors that are directly or indirectly linked to agriculture are affected, it simply means that the agricultural sector is also affected. This effect on the agricultural sector will make it difficult for the country to achieve its SDG targets. Edame et al. (2011) argued that extremely intense weather will have unforeseen impacts on food production and distribution infrastructure on livelihoods of rural and urban dwellers. Also, changes in mean temperature and rainfall will strongly affect the suitability of land for different types of crops. The health and productivity of forests and the incidence of pests and diseases, biodiversity and ecosystems will also be seriously affected. In that regard, achieving the SDG 2 will be a challenging mission.

Mpandeli et al. (2005) discovered that the rural community of Limpopo Province grew maize and vegetables. However, adverse climatic conditions will have a bearing on their agricultural production. Ziervogel (2014) asserted that the impact of climate change on agriculture will most likely be on staple crops such as maize and rice. One of the chief concerns is a projected increase in irrigation because most parts of the country are likely to become drier. Moreover, another concern is in line with certain pest and pathogen species which are likely to benefit from the change in climate and ultimately become a problem for agriculture. In this regard, SDG number 2.4 asserts that:

by 2030 ensure sustainable food production systems and implement resilient agricultural practices that increase productivity and production, that help maintain ecosystems that strengthen capacity for adaptation to climate change, extreme weather, drought, flooding and other disasters and that progressively improve land and soil quality. (ICSU & ISSC 2015:6)

Hence, the effect of climate change on staple crops is double headed in an effort to achieve SDG 2. Firstly, agriculture will be affected, and as a result, staple crops will result in failure to end hunger and malnutrition because majority of South Africans rely on these staple crops for their day-to-day survival. Secondly, because certain pest and pathogen species will benefit from the changing climate, subsistence farmers will likely not be able to access required pesticides because of lack of funds. Hence, agricultural production will be significantly disturbed and that will lead to increased hunger and malnutrition.

Furthermore, Madzwamuse (2010) discovered that increasing temperatures and reduced rainfall will collectively impact the agricultural systems in South Africa. Considerable impacts include reduction in the amount of land appropriate for both arable and pastoral agriculture. The reduction in the size of the growing season and a decrease in yields mostly along the margins of semi-arid and arid areas account for the substantial impacts of climate change. Climate change is likely to reduce the role of agriculture to the GDP, which has been on the verge of declining over the years. In 1998, agriculture and forestry added 4.0% to the GDP, which is ominously lower than the 9.1% recorded in 1965 (NDA 2000). Reduced rainfall has negative impacts on large-scale agriculture, which relies on irrigation and the rural poor who practice rain-fed agriculture. These impacts have extensive consequences for national food security and economy. Consequently, projected impacts of water shortages will likely affect yield production of various crops and the productivity of rangelands by 2050. Because the country largely relies on large-scale farmers for food products, disrupted agricultural productivity because of climate change will seriously affect accessibility and affordability of food products owing to food shortages. Consequently, this will also result in hunger and malnutrition. Moreover, if GDP continues to be affected, it means poverty and hunger will increase and achieving SDG 2 will be a challenging objective for South Africa.

## Conclusion

As a developing country, South Africa faces a daunting task of working towards achieving SDG 2. The failure to meet the 2015 target of the MDGs on ending hunger was a result of failure by the governments of developing countries to make commitments directed at curbing the major barriers to the targets. For instance, in order to end hunger in South Africa, progressive agricultural policies that provide knowledge and relevant equipment to poor subsistence farmers in rural communities must be put in place. This will act as one way of adaptation to climate change in rural communities. South Africa is on the path of development and its rural communities are immensely vulnerable to natural disasters and climatic hazards owing to the lack of knowledge and technology to deal with the effects of climate change. Moreover, the effects of climate change on agriculture significantly results in increased hunger and malnutrition. Hence, achieving SDG 2 in the midst of climate change seems to be a serious challenge for South Africa.

### Recommendations

Achieving SDG 2 would mean that the South African government needs to take into consideration certain pertinent aspects. These include forging progressive agricultural policies that are vehemently inclusive as well as ensuring that all people are aware of climate change and its effects as well as adaptation methods.

### Progressive agricultural policies

The government should implement progressive agricultural policies that empower subsistence farmers in rural communities so that they enhance agricultural productivity in the midst of climate change. This will reduce the high levels of hunger and malnutrition which usually result from vulnerable rural communities.

### Knowledge of climate change

People in rural communities should be taught about climate change and its possible effects on their activities. They should also be imparted with adaptation methods, that is, knowledge on how to manage their activities in the presence of climate change.

### Climate-smart policies

The government through the ministry of agriculture should develop climate-smart agriculture policies in order to enhance small holder farmers’ adaptation to climate change.
